# Diagnostic Yield and Clinical Impact of a Small Genetic Panel for Kidney Disease: A Multicenter, Retrospective European Study

**DOI:** 10.1111/cge.70002

**Published:** 2025-06-18

**Authors:** Silvia Giovanella, Antonio Miguel Poyatos‐Andújar, Maria Mar Aguila Garcia, Almudena Avila‐Fernandez, Ana Bustamante‐Aragonés, Carmen Ayuso, Antonio Percesepe, Davide Martorana, Maria Ferri, Alessandra Terracciano, Laura Massella, Johanna Chester, Francesca Testa, Giulia Ligabue, Marco Ferrarini, Dino Gibertoni, Gaetano Alfano, Elena Tenedini, Lucia Artuso, Marco Marino, Olga Calabrese, Enrico Tagliafico, Riccardo Magistroni

**Affiliations:** ^1^ Department CHIMOMO University of Modena and Reggio Emilia Modena Italy; ^2^ Unidad de Gestión Clínica de Laboratorios, GEEPAD, Hospital Universitario Virgen de las Nieves Granada Spain; ^3^ Department of Genetics & Genomics Centre for Biomedical Network Research on Rare Diseases Madrid Spain; ^4^ Department of Surgery and Medicine University Hospital Parma Italy; ^5^ UO Genetica Medica‐University Hospital Parma Italy; ^6^ University Hospital of Modena, Nephrology, Dialysis and Kidney Transplantation Modena Italy; ^7^ Laboratory of Medical Genetics Translational Cytogenomics Research Unit, Bambino Gesù Children's Hospital, IRCCS Rome Italy; ^8^ Division of Nephrology Bambino Gesù di Roma Children's Hospital, IRCSS Rome Italy; ^9^ IRCCS University Hospital of Bologna, Epidemiology and Statistics Unit Bologna Italy; ^10^ Department of Medical and Surgical Sciences University of Modena and Reggio Emilia, University Hospital of Modena, Diagnostic Hematology and Clinical Genomics Unit Modena Italy; ^11^ University Hospital of Modena, Diagnostic Hematology and Clinical Genomics Unit Modena Italy; ^12^ University Hospital of Modena, Medical Genetics Modena Italy

**Keywords:** CKD, clinical impact, clinical predictors, diagnostic yield, genetic panel

## Abstract

Chronic kidney disease (CKD) has a genetic origin in 10% of patients. The most effective and cost‐beneficial genetic testing methodology is debated. A multicenter, retrospective analysis of 692 patients with panel genetic testing (44 genes) evaluated the diagnostic yield, independent predictors of genetic diagnoses, and clinical impact. Diagnostic variants identified totaled 252, resulting in a 36% yield. The highest yields were associated with cystic disease (49%). No diagnostic variants were identified in unknown CKD. Independent clinical predictors of diagnosis were clinical presentation, family history, and early disease onset. Genetic diagnoses confirmed clinical suspicion in 70%, defined the diagnosis in 23%, and altered clinical diagnosis in 7%. Despite study limitations, a 44 gene panel seems to have a similar diagnostic yield as larger panels and whole‐exome sequencing (WES) approaches. Patient selection based on independent predictors of genetic diagnosis may further increase diagnostic yield and cost‐effectiveness, especially useful in cost‐restricted contexts.

## Graphical abstract

In a multicenter study of 692 patients with kidney disease, a 44‐gene panel achieved a 36% diagnostic yield. The 36% yield was not significantly lower than with larger panels or whole‐exome sequencing. A post hoc machine‐learning model identified key predictors to stratify patients into four risk bins (11%–74% yield), enabling more cost‐effective testing.

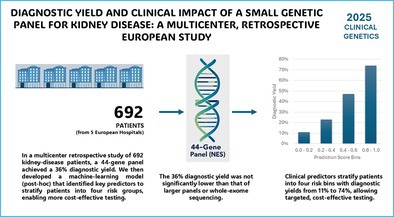

## 1 Introduction

In an unselected population with chronic kidney disease (CKD), it is estimated that a genetic cause is present in approximately 10% of cases [1]. Furthermore, it has recently been reported that approximately 10% of adult kidney failure (KF) is attributable to genetic variants [2, 3]. There are several approaches to genomic analysis, the diagnostic yield of these technologies varies considerably, mainly due to methodological inter‐study heterogeneity in cohort, patient selection, and genetic testing [4].

Panel genetic testing is a well‐established, cost‐effective technology [5], but with some limitations, including a restricted number of genes. An unrestricted exome‐wide approach as WES can outperform panels in diagnostic yield for complex phenotypes, but targeted panels may be equally or more effective for well‐defined conditions, depending on clinical context and resources. Studies show whole‐genome sequencing (WGS) can increase diagnostic yield by ~2%–9% over WES [6, 7, 8]. However, WGS raises ethical and financial concerns that may not justify the modest diagnostic gains [9, 10, 11].

We hypothesize that a small, targeted nephropathy panel has a comparable diagnostic yield to larger genetic panels and WES. In this retrospective multicenter study, we aim to report the diagnostic yield of a targeted panel of 44 genes and clinical predictive factors of diagnoses.

## 2 Materials and Method

### 2.1 Study Design and Patient Cohort

This is a retrospective, multicenter study which aims to report the diagnostic yield of a genetic panel across five European centers. Patients were not selected according to a formal, prospectively defined set of inclusion criteria. Instead, the study cohort consists of patients who underwent the NES (nephropathy solution) genetic test between 2018 and 2023 at the participating centers as part of routine clinical care. The decision to pursue genetic testing was made by the attending physicians based on clinical judgment. The clinical impact study focused on participants enrolled in the coordinating center (University Hospital, Modena).

Pre‐test clinical presentation was classified into seven clinical categories (Table S1). Demographic (age, sex) and clinical (age at genetic test, symptom onset and KF, kidney function, and family history) information were collected.

The Ethics Committee of the coordinating center approved the study (Prot.0036074/21), and each participating center had local ethics approval. The study was conducted according to the Declaration of Helsinki. The STROBE guidelines for reporting observational studies (STROBE) were followed.

### 2.2 Multigene NGS Panel Sequencing

The NES panel is a 44 gene commercial test developed by SOPHiA GENETICS (further details in Table S2). Genetic analysis was performed via the Illumina MiSeq platform (performance metrics in Supporting Information). Sequencing data were processed for single nucleotide variants (SNVs), indels, and copy number variations (CNVs). Diagnostic variants were defined according to the American College of Medical Genetics and Genomics (ACMG) guidelines [12]. Cases in which only a single pathogenic or likely pathogenic (C5/C4) variant was identified (monoallelic) in autosomal‐recessive kidney disease genes are detailed in the Supporting Information (see Tables S4–S8).

### 2.3 Predictive Diagnostic Algorithm and Statistical Analysis

A machine‐learning approach was employed to predict positive genetic diagnoses (additional details in Supporting Information: machine‐learning analysis to predict positive genetic test).

The diagnostic yield was calculated as the number of diagnostic variants found to the total variants and expressed as a percentage. Quantitative data are shown as median and interquartile range (IQR). Categorical variables are expressed as percentages.

## 3 Results

### 3.1 Cohort Characteristics

A total of 809 cases with genetic testing were enrolled from the five participating centers. Subjects who underwent targeted cascade testing (117 cases) were excluded from further analysis. The study cohort (692 index cases) characteristics are outlined in Table S3. A positive family history of kidney disease was reported by 57% of the subjects. The most prevalent clinical presentation was cystic disease (54%, see Table 1).

**TABLE 1:** Overall and clinical presentation–specific diagnostic variants and yields of the main population cohort, as assessed with a targeted diagnostic panel.

Clinical presentation, *n (%)*	Patients, *n* (n/N)	Diagnostic variants observed^a^, *n*	Diagnostic yield, %	95% CI (%)	Most frequently observed gene (%)
Cystic disease	371 (54)	183	49	44.2–54.4	PKD1 (64)
Glomerulopathy	184 (27)	52	28	21.8–34.8	COL4A5 (40)
CAKUT	45 (6)	6	13	3.4–23.2	HNF1b (50)
Nephrolithiasis	36 (5)	4	11	0.8–21.4	CYP24A1 (100)
Tubulopathy	22 (3)	7	32	12.3–51.3	SLC12A3 (50)
uCKD	22 (2)	0	0	0–15.4	N/A
At risk—negative phenotype	12 (2)	0	0	0–25	N/A
Total	692 (100)	252	36	32.4–39.6	PKD1 (47)

Abbreviations: CAKUT, congenital abnormalities of kidney and urinary tract; CI, confidence interval; N/A, not applicable; uCKD, unknown chronic kidney disease.

^a^ Diagnostic variants comprise “pathogenic/C5” and “likely pathogenic/C4” according to the American College of Medical Genetics and Genomics (ACMG) guidelines [12].

### 3.2 Variant Analysis and Diagnostic Yield

The total diagnostic yield was 36% (*n* = 252, see Table 1). All diagnostic variants are outlined in Tables S4–S8. Among the diagnostic variants identified, 8 genes accounted for 95% of the diagnoses: PKD1 (47%), PKD2 (21%), COL4A5 (9%), COL4A3 (7%), COL4A4 (4%), PKHD1 (4%), SLC12A3 (2%), and CYP24A1 (2%).

### 3.3 Clinical Predictors of Genetic Diagnosis

The predictive algorithm for genetic diagnosis likelihood achieved an AUC‐ROC of 0.78, sensitivity (TPR) of 0.55 and precision of 0.71 (Table S9).

Four factors were identified as independent predictors of effect: clinical presentation (OR 6.2, 95% CI 2.3–21.5, cystic disease vs. nephrolithiasis), family history (OR 4.7, 95% CI 3.2–7.2), early disease onset (OR 2.2, 95% CI 1.5–3.3), and KF (OR 1.6, 95% CI 1.1–2.5). The calculator is freely available online: https://decide‐xxej.onrender.com/.

### 3.4 Clinical Impact

**FIGURE 1:**
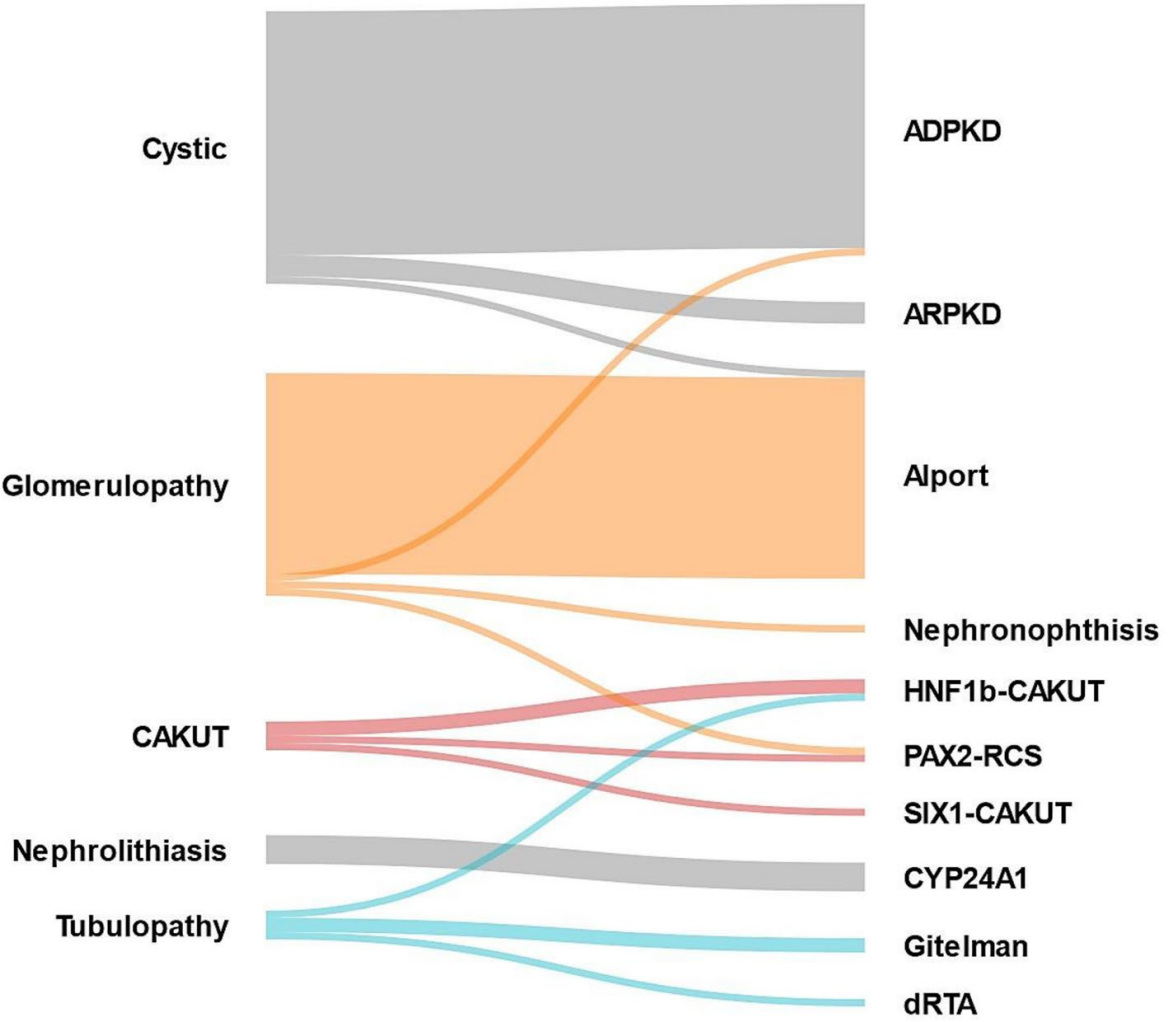
A Sankey diagram for clinical diagnosis (left) and genetic diagnosis (right). ADPKD, autosomal dominant polycystic kidney disease; ARPKD, autosomal recessive polycystic kidney disease; CAKUT, congenital abnormalities of kidney and urinary tract; dRTA, distal renal tubular acidosis; FSGS, focal segmental glomerulosclerosis; RCS, renal coloboma syndrome.

Patients with diagnostic genetic tests enrolled at the coordinating center (*n* = 86), were assessed for clinical impact. The clinical diagnosis was confirmed in 70% (*n* = 60), established in 23% (*n* = 20) without a pre‐test clinical diagnosis, and changed in 7% (*n* = 6) (see Figure 1 and Table S10). In several cases, genetic testing had a direct influence on clinical management. For instance, reclassifying an initially undefined case as an HNF1B‐related disease led to focused monitoring for diabetes and tailored reproductive counseling while confirming Alport syndrome in other patients spared unnecessary immunosuppressive treatment and prompted targeted hearing and vision surveillance. A detailed report about the clinical impact is reported in Supporting Information.

## 4 Discussion

The present study reports a 36% yield for a 44‐gene panel in 692 patients with suspected genetic kidney disease. Predictive factors included clinical presentation, family history, early disease onset, and KF. Clinical impact revealed confirmation of clinical diagnosis in 70%, new diagnosis in 23%, and changed in 7%.

These findings highlight the diagnostic potential of targeted genetic panels for kidney conditions. Implementation and cost‐effectiveness remain critical, especially in primary nephrology centers with limited resources. Our study's 36% diagnostic yield with 44 genes is comparable to studies using large panels and WES [4]. Disease‐specific panels for adults range from 7% to 22% [13, 14, 15], whereas broader nonspecific panels yield 12%–50% [16, 17]. Two recent studies by Bleyer et al. [18] and Dahl et al. [5] using a 385‐gene panel reported a 21% diagnostic yield in an unselected CKD population. However, comparing yields is complex due to heterogeneous populations. A high number of patients in the present study had predictive factors for suspected genetic diseases: 57% with family history compared with 36% in Dahl et al. The clinical decision to request genetic testing in cases of high suspicion of genetic disease can be expected to influence the diagnostic rate compared with testing in an unselected CKD population.

Consistent with other reports, we found that a small number of genes dominate the genetic landscape of CKD. Five genes (PKD1, PKD2, and COL4A3/A4/A5) accounted for the majority of diagnoses in both our cohort and others [1, 5, 18], suggesting that focusing on these common genes can capture most hereditary kidney disease cases. Because of the peculiar epidemiology of the kidney Mendelian conditions, we speculate that a small epidemiologically‐based panel may offer a cost‐effective initial screening alternative.

We described an algorithm to predict diagnosis likelihood with the NES panel, useful for uncertain cases. Our additional bin‐based analysis (in Supporting Information) provides practical evidence of how the model's output can inform clinical decisions. Nonetheless, we acknowledge that larger prospective studies are needed to confirm these findings and establish robust cut‐off thresholds for routine clinical use.

Several important limitations must be acknowledged. First, the 44‐gene panel cannot detect variants in genes beyond its scope. Broader sequencing (WES/WGS) can uncover those rare causes, though these methods come with higher costs, ethical concerns, and more incidental findings that may limit their routine use. Second, this was a retrospective study without prospective validation of the model or direct comparison to broader sequencing. Thus, our findings should be interpreted with caution until confirmed by prospective studies or external validation. Third, variations in workup and incomplete phenotypic data across centers could have influenced our diagnostic yield. Future studies should employ standardized clinical protocols, enroll patients prospectively at multiple centers, and directly compare different genetic testing strategies.

Despite these limitations, our data suggest that a small, well‐designed panel remains a cost‐effective screening tool for common forms of genetic kidney disease, particularly in resource‐limited contexts or when clinical suspicion strongly indicates one of the most prevalent genes (e.g., PKD1, PKD2, or COL4A). A prospective, multicenter study comparing small panels with WES/WGS approaches would help clarify the optimal balance between breadth of coverage, cost, and clinical utility.

## Author Contributions

All authors collected genetic and/or clinical data from the five centers involved and revised the article. S.G. and R.M. drafted the article. All authors approved the article.

## Conflicts of Interest

The authors declare no conflicts of interest.

## Supporting information


**Data S1.** cge70002‐sup‐0001‐Supinfo.

## Data Availability

Data supporting this study are included within the article and Supporting Information.
