# Supplementary material for: Diagnostic Yield and Clinical Impact of a Small Genetic Panel for Kidney Disease: A Multicenter, Retrospective European Study
Source: Clin Genet. 2025 Jun 18;109(1):136–40. doi: 10.1111/cge.70002 (PMC12674980; doi:10.1111/cge.70002)
Supplement: Supplementary file 1 — Data S1. cge70002‐sup‐0001‐Supinfo. [file CGE-109-136-s001.docx]

**SUPPLEMENTARY MATERIAL**

DIAGNOSTIC YIELD AND CLINICAL IMPACT OF A SMALL GENETIC PANEL FOR KIDNEY DISEASE: A MULTICENTER, RETROSPECTIVE EUROPEAN STUDY

Silvia Giovanella^1^, Antonio M. Poyatos Andújar^2^, Maria Mar Aguila Garcia^2^, Almudena Avila-Fernandez^3^, Ana Bustamante-Aragones^3^, Carmen Ayuso^3^_,_ Antonio Percesepe^4,5^, Davide Martorana^5^, Maria Ferri^6^, Alessandra Terraciano^7^, Laura Massella^8^, Johanna Chester^1^, Francesca Testa^6^, Giulia Ligabue^1^, Marco Ferrarini^1^, Dino Gibertoni^9^, Gaetano Alfano^6^, Elena Tenedini^10^, Lucia Artuso^10^, Marco Marino^11^, Olga Calabrese^12^, Enrico Tagliafico^10^, Riccardo Magistroni^1,6*^

**Performance metrics of the test** Pag. 2

**Machine learning analysis to predict positive genetic test** Pag. 2

**Further genetic investigations in patients with negative NES panel** Pag. 2

**Stratifying Diagnostic Yield Using Machine Learning Predicted Probabilities**

**for Cost-Effective Genetic Testing Prioritization** Pag. 3

**Clinical impact of the genetic test** Pag. 5

**Diagnostic Implications of Single Heterozygous Variants in**

**Autosomal-Recessive Kidney Disorders** Pag. 6

**Supplementary Tables**

**Table S1:** Definitions of clinical presentations. Pag. 7

**Table S2:** Genes included in the commercially available panel (NES) and the related diseases. Pag. 7

**Table S3:** Demographic and clinical characteristics of the main population cohort, grouped according to clinical diagnoses. Pag. 10

**Table S4:** Diagnostic variants in patients with Cystic disease. Pag. 11

**Table S5:** Diagnostic variants in patients with Glomerulopathy. Pag. 15

**Table S6:** Diagnostic variants in patients with CAKUT. Pag. 17

**Table S7:** Diagnostic variants in patients with Nephrolithiasis. Pag. 18

**Table S8:** Diagnostic variants in patients with Tubulopathy. Pag. 18

**Table S9:** Predictive model evaluation score based on the test set (accuracy, area under the receiver operating characteristic curve (AUC), F1 score, precision, recall, and specificity). Pag. 19

**Table S10:** Reclassification of the initial clinical suspicion, after genetic testing. Pag. 19

**Performance metrics of the NES Panel**

The target coverage (coding DNA sequencing and splicing sites) is, for all genes, 100%.

NGS sensitivity, specificity and accuracy: >99%.

Reproducibility: >99%.

Repeatability: >99%.

The sequencing mean depth for target genes is > 400X with a minimum depth of 100X in 100% of covered regions.

Pathogenic and likely pathogenic single nucleotide variants (SNVs) and Indels were confirmed by Sanger sequencing and copy number variations (CNVs) with multiplex ligation-dependent probe amplification (MLPA).

**Machine learning analysis to predict positive genetic test**

A predictive model for genetic diagnosis likelihood was developed using patient-specific clinical data: sex, clinical presentation, age at onset, age at genetic test, early disease onset, kidney function, hypertension, kidney failure, age at renal replacement therapy initiation and family history. The model was developed using the PyCaret classification module (PyCaret package - https://github.com/pycaret/pycaret/releases). Missing data were handled using mean imputation. The best predictive algorithm was selected based on the highest area under the receiver operating characteristic curve (AUC-ROC). Internal validation was carried out using a stratified 10-fold cross-validation approach, ensuring that the distribution of positive and negative cases was maintained across folds. The subjects were randomly assigned to training (70%) or test (30%) sets. The final model was evaluated on the independent hold-out test set (30% of the data). The performance metrics reported in the manuscript—such as accuracy, AUC-ROC, sensitivity, specificity, precision, and F1 score—refer to the final test set evaluation.

**Further genetic investigations in patients with negative NES panel**

Further investigations were conducted in 25 patients with negative NES panel analyses. In 3 cases (12%), a genetic diagnosis was established via WES (n=2; PKD1: c.10167+25_10167+43del, NPHP1 all exons homozygous deletion – MLPA validated) and larger targeted panels (n=1; INF2: p.Glu220Lys,).

**Stratifying Diagnostic Yield Using Machine Learning Predicted Probabilities for Cost-Effective Genetic Testing Prioritization**

To further illustrate how our model can guide clinical decision-making, we stratified the test set into four bins of predicted probability: 0.0–0.2, 0.2–0.4, 0.4–0.6, and 0.6–1.0. In these groups, the diagnostic yield was 11.1%, 23.1%, 47.1%, and 73.7%, respectively (Supplementary Figures S1 and S2). This clear gradient indicates that patients with higher predicted scores have a substantially increased likelihood of harboring a pathogenic variant. Consequently, in clinical practice, individuals in the highest bin (≥0.6) may be prioritized for genetic testing to confirm or refine diagnosis, whereas those with lower scores can be tested subsequently or monitored closely until further clinical evidence emerges. This approach enables a more cost-effective and targeted allocation of genetic testing resources.

**Figure S1:** Diagnostic yield across predicted score bins.

The test set was stratified into four predicted probability bins (0.0–0.2, 0.2–0.4, 0.4–0.6, and 0.6–1.0). The diagnostic yield increased progressively across bins, ranging from 11.1% in the lowest bin to 73.7% in the highest. This trend suggests that higher predicted scores correspond to an increased likelihood of detecting a pathogenic variant.

**
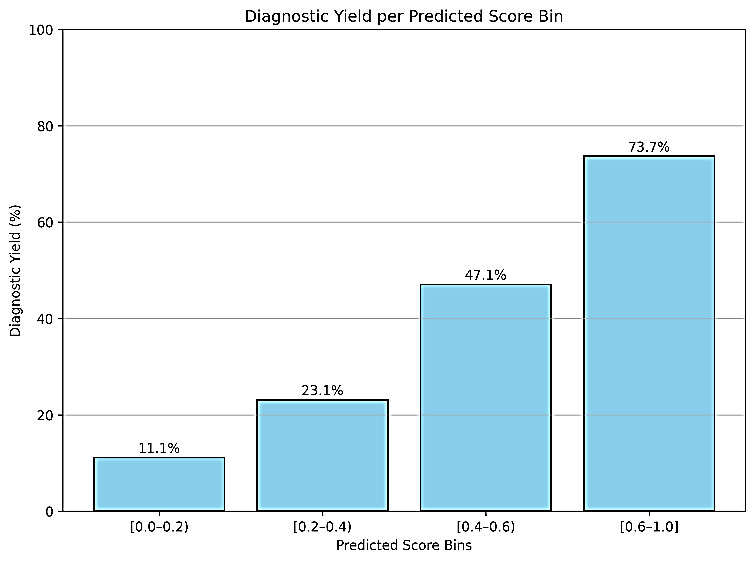
**

**Figure S2:** Distribution of predicted scores with diagnostic outcomes.
The distribution of predicted scores is shown in a stacked bar format, distinguishing diagnostic (blue) and non-diagnostic (gray) cases within each bin. The proportion of diagnostic cases increases in higher predicted score bins, supporting the clinical utility of the predictive model for guiding genetic testing prioritization.

**
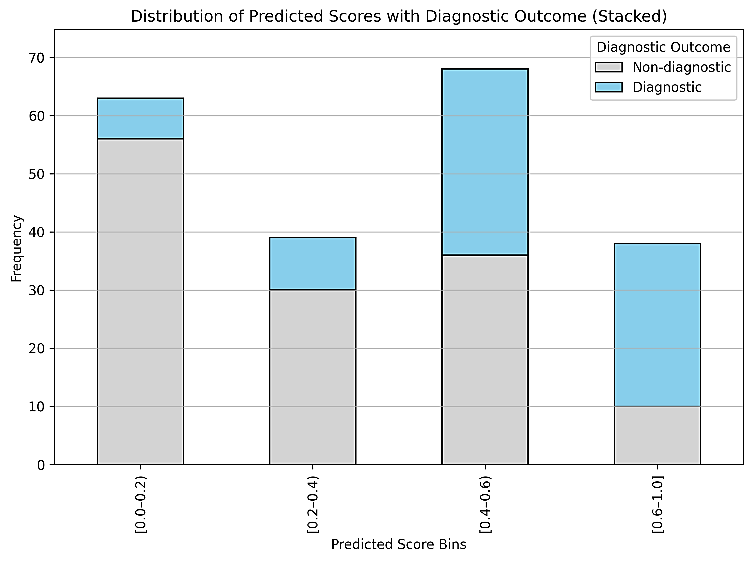
**

**Clinical impact of the genetic test**

Confirmation of clinical diagnoses included cystic disease (n=46), Alport syndrome (n=12), and Gitelman syndrome (n=2). New established diagnoses included Alport syndrome (n=15) presented by a previous undiagnostic kidney biopsy or urine abnormalities, autosomal recessive polycystic kidney disease (ARPKD) (n=1), Nephrolithiasis (n=1), distal renal tubular acidosis (n=1), HNF1b-CAKUT (n=1), Imerslund-Grasbeck syndrome (n=1). Changed diagnoses included autosomal dominant polycystic kidney disease (ADPKD) (n=1), Alport syndrome (n=2), Nephronophthisis (n=1), Renal Coloboma Syndrome (n=1) and HNF1b-CAKUT (n=1) (Figure 1, Supplementary Table S10).

Genetic diagnosis led to individualized follow-up and surveillance in most cases (n=63). Patients were counseled for diabetes (HNF1b), cerebral aneurysm (ADPKD), coloboma (PAX2) and hearing loss (COL4A5 and ATP6V0A4). In 18 cases, patients were enrolled in national registries, and 6 cases were assessed for clinical trials. Unnecessary renal biopsy and futile immunosuppressive treatments were successfully avoided in 10 cases. Familial eligibility for living donor kidney transplantation was performed in 3 cases (transplants were successfully undertaken), and reproductive counseling was offered in 6 cases. Genetic testing was extended to family members with early disease identification in 21 cases.

**Diagnostic Implications of Monoallelic Variants in Autosomal-Recessive Kidney Disorders**

In our cohort, 13 patients across six distinct autosomal-recessive renal conditions—PKHD1-related ARPKD (n = 4), CYP24A1-related idiopathic infantile hypercalcemia/nephrolithiasis (n = 4), SLC12A3-related Gitelman syndrome (n = 2), SLC4A4-related proximal renal tubular acidosis (n = 1), ATP6V0A4-related distal renal tubular acidosis (n = 1), and GRHPR-related primary hyperoxaluria (n = 1)—presented with a classical phenotype despite harboring only a single pathogenic or likely pathogenic (ACMG C5/C4) variant.

According to ACMG/AMP recommendations, a definitive diagnosis of an autosomal recessive disorder ordinarily requires pathogenic variants on both alleles (Richards et al., 2015). While identification of a single pathogenic variant in a gene associated with an autosomal recessive disorder can offer valuable diagnostic insight in patients exhibiting a highly specific phenotype, it does not satisfy the ACMG/AMP requirement for two pathogenic alleles and must therefore be interpreted with caution in the broader context of clinical and genetic data. Our NES panel uses read-depth and split-read algorithms to call exon-level CNVs, with demonstrated sensitivity for PKD1 and other genes. Nonetheless, small/deep intronic/promoter CNVs or complex rearrangements may remain undetected by standard pipelines.

Burgmaier et al. have documented that even PKHD1 obligate heterozygotes can exhibit subclinical imaging abnormalities such as increased medullary echogenicity or small liver cysts. Furthermore, these authors have emphasized that, even in confirmed biallelic cases, detecting deep-intronic variants or complex rearrangements often requires whole-genome sequencing beyond standard panel-based CNV pipelines. Similarly, CYP24A1 carriers may show altered vitamin D homeostasis with the potential risk of developing hypercalcemia and related clinical manifestations if exposed to triggering factors (Brancatella et al., 2021). Such findings suggest that a single pathogenic variant, in the presence of a highly specific phenotype, should not be summarily dismissed. In this framework, monoallelic findings generally warrant cautious interpretation and, where possible, additional investigations to identify a second pathogenic allele.

1. Richards S, Aziz N, Bale S, et al. Standards and guidelines for the interpretation of sequence variants: a joint consensus recommendation of the American College of Medical Genetics and Genomics and the Association for Molecular Pathology. Genetics in medicine : official journal of the American College of Medical Genetics. 2015;17(5):405-424.
2. Burgmaier K, Gimpel C, Schaefer F, et al. Autosomal Recessive Polycystic Kidney Disease – PKHD1. 2001 Jul 19 [Updated 2024 Apr 4]. In: Adam MP, Feldman J, Mirzaa GM, et al., editors. GeneReviews®. Seattle (WA): University of Washington, Seattle; 1993-2025. Available from: <https://www.ncbi.nlm.nih.gov/books/NBK1326/>
3. Brancatella A, Cappellani D, Kaufmann M, et al. Do the Heterozygous Carriers of a CYP24A1 Mutation Display a Different Biochemical Phenotype Than Wild Types? *The Journal of clinical endocrinology and metabolism.* 2021;106(3):708-717.

**Table S1:** Definitions of clinical presentations.

|  | **Definitions** |
| --- | --- |
| Cystic kidney disease | presence of renal cysts by radiologic examination with or without a positive family history |
| Glomerulopathy | urine abnormalities with or without chronic kidney disease. In the case of an available renal biopsy, the diagnosis must not be conclusive |
| CAKUT | congenital abnormalities of kidney and urinary tract, hyperechoic hypo-dysplastic kidneys |
| Tubulopathy | dysfunction in specialized channels and transporters (glycosuria, aminoaciduria, polyuria, bicarbonaturia, increased beta2-microglobinuria, proteinuria, hypercalciuria, renal tubular acidosis, etc.) |
| Nephrocalcinosis/Nephrolithiasis | renal stones, renal calcifications, nephrocalcinosis |
| At risk with negative phenotype | negative renal phenotype but affected relatives with a likely autosomal dominant disease segregation. (e.g. kidney donation assessment) |
| Unknown CKD | undiagnosed CKD or late referral |

*CAKUT*, congenital abnormalities of kidney and urinary tract; *CKD*, chronic kidney disease.

**Table S2:** Genes included in the commercially available panel (NES, Nephropathies Solution, SOPHiA GENETICS, Switzerland) and the related diseases.

| **Genes** | **Transcript references** | **Disease** |
| --- | --- | --- |
| AGXT | NM_000030.2 | Primary Oxaluria type 1 |
| AQP2 | NM_000486.5 | Nephrogenic diabetes insipidus |
| ATP6V0A4 | NM_020632.2 | Renal tubular acidosis |
| ATP6V1B1 | NM_001692.4 | Renal tubular acidosis with deafness |
| AVPR2 | NM_000054.6 | Nephrogenic diabetes insipidus |
| BSND | NM_057176.2 | Bartter syndrome, type 4 |
| CASR | NM_000388.3 | Hypocalcemia |
| CEP290 | NM_025114.3 | Joubert syndrome |
| CLCN5 | NM_001127898.3 | Dent Disease |
| CLCNKB | NM_000085.4 | Bartter syndrome, type 3 |
| COL4A3 | NM_000091.4 | Alport syndrome |
| COL4A4 | NM_000092.4 | Alport syndrome |
| COL4A5 | NM_000495.3 | Alport syndrome |
| CRB2 | NM_173689.7 | Focal segmental glomerulosclerosis |
| CTNS | NM_001031681.2 | Cystinosis |
| CUBN | NM_001081.3 | Megaloblastic anemia-1, Finnish type |
| CYP24A1 | NM_000782.4 | Hypercalcemia, infantile |
| DSTYK | NM_015375.3 | CAKUT |
| EMP2 | NM_001424.5 | Nephrotic syndrome |
| EYA1 | NM_000503.5 | Branchiootic syndrome |
| FN1 | NM_002026.3 | Glomerulopathy with fibronectin deposits |
| FOXC1 | NM_001453.2 | Dysgenesis |
| GRHPR | NM_012203.2 | Hyperoxaluria, primary type II |
| HNF1b | NM_000458.3 | ADTKD |
| KANK2 | NM_001136191.2 | Nephrotic syndrome, type 16 |
| KCNJ1 | NM_000220.4 | Bartter syndrome, type 2 |
| LAMB2 | NM_002292.4 | Nephrotic syndrome, type 5 |
| NPHS2 | NM_014625.3 | Nephrotic syndrome, type 2 |
| NR3C2 | NM_000901.4 | Pseudohypoaldosteronism type I |
| OCRL | NM_000276.3 | Dent disease 2 |
| PAX2 | NM_000278.4 | Focal segmental glomerulosclerosis |
| PHEX | NM_000444.5 | Hypophosphatemic rickets |
| PKD1 | NM_001009944.2 | ADPKD |
| PKD2 | NM_000297.3 | ADPKD |
| PKHD1 | NM_138694.3 | ARPKD |
| SIX1 | NM_005982.4 | Branchiootic syndrome 3 |
| SLC12A1 | NM_000338.2 | Bartter syndrome, type 1 |
| SLC12A3 | NM_000339.2 | Gitelman syndrome |
| SLC34A1 | NM_001167579.1 | Hypercalcemia infantile |
| SLC4A1 | NM_000342.3 | Renal tubular acidosis |
| SLC4A4 | NM_001098484.3 | Renal tubular acidosis |
| TTC21B | NM_024753.4 | Nephronophthisis |
| UMOD | NM_001008389.2 | ADTKD |
| WT1 | NM_000378.4 | Denys-Drash syndrome |

*CAKUT*, congenital abnormalities of kidney and urinary tract; *ADTKD*, Autosomal dominant tubulointerstitial kidney disease; *ADPKD*, Autosomal dominant polycystic kidney disease; *ARPKD* Autosomal recessive polycystic kidney disease.

The gene content of this panel was determined by the manufacturer, who selected 44 genes based on an extensive review of the literature, epidemiological data, and clinical utility in nephropathies. This panel is designed to maximize diagnostic yield while maintaining cost-effectiveness and a rapid turnaround time. The NES panel is routinely used as the initial screening tool owing to its rapid turnaround time, high gene coverage, and robust performance in complex regions such as PKD1. While not free, the panel is more cost-effective than WES, thereby optimizing resource allocation in clinical practice. However, due to its commercial nature, the panel does not include every gene associated with kidney disease. For instance, certain genes relevant to cystic kidney disease (such as IFT140), some genes associated with autosomal dominant polycystic liver disease (ADPLD), and additional genes implicated in autosomal dominant tubulointerstitial kidney disease (ADTKD) are not represented. We acknowledge that this limitation may affect the detection of rare or atypical variants, and we recommend that in cases with high clinical suspicion but negative panel results, additional comprehensive genomic testing (e.g., WES or WGS) may be warranted.

**Table S3:** Demographic and clinical characteristics of the main population cohort, grouped according to clinical diagnoses.

| **Characteristics** | **Total cohort  (N=692, 100%)** | **Cystic disease (n=371, 54%)** | **Glomerulopathy  (n=184, 27%)** | **CAKUT  (n=45, 6%)** | **Nephrolithiasis  (n=36, 5%)** | **Tubulopathy  (n=22, 3%)** | **uCKD  (n=22, 3%)** | **At risk-negative phenotype  (n=12^#^, 2%)** |
| --- | --- | --- | --- | --- | --- | --- | --- | --- |
| **Demographics** |  |  |  |  |  |  |  |  |
| Female, *n (%)* | 263 (45) | 116 (42) | 91 (51) | 19 (43) | 14 (39) | 11 (55) | 6 (19) | 6 (50) |
| Family history, *n (%)*^a^ | 311 (57) | 161 (61) | 86 (55) | 13 (32) | 22 (73) | 6 (31) | 11 (58) | 12 (100) |
| Age at symptoms onset, *yr* | 38 (23-51)† | 40 (24-51)‡ | 40 (24-55) | 23 (3-41)†‡ | 38 (18-47) | 35 (21-54) | 33 (24-49) | N/A |
| Age at genetic test, *yr* | 50 (38-61) | 50 (39-62) | 55 (41-62) | 37 (28-47) | 48 (39-57) | 46 (30-58) | 51 (39-59) | 41 (29-52) |
| **Clinical (at presentation)** |  |  |  |  |  |  |  |  |
| eGFR, *ml/min per 1,73m^2^* | 64 (41-95) | 70 (43-98) | 55 (38-88) | 60 (38-79) | 81 (55-99) | 56 (41-81) | 42 (18-50)† | 100 (90-109)† |
| Hypertension, *n (%)* | 350 (66) | 163 (64) | 132 (79) | 23 (59) | 13 (50) | 4 (22)† | 15 (83)† | 0 |
| Kidney failure, *n (%)* | 112 (19) | 41 (15) | 45 (26) | 8 (18) | 4 (11) | 0† | 14 (64)† | 0 |
| Age of Kidney failure, *yr* | 46 (32-59) | 50 (42-60) | 47 (29-61) | 31 (23-50) | 48 (33-72) | N/A | 37 (26-45) | N/A |

Unless otherwise indicated, values are presented as median (25th–75th percentile). GFR was estimated according to the Chronic Kidney Disease Epidemiology Collaboration formula.

*CAKUT*, congenital abnormalities of kidney and urinary tract; *uCKD*, Unknown Chronic Kidney Disease, *N/A*, not applicable.

^a^ Data available for 539 patients. **^#^** Other patients at risk were tested with targeted tests and excluded from the analysis.

†, ‡ Pairwise statistically significant, *P* < 0.001

**Table S4:** Diagnostic variants in patients with Cystic disease.

| **PatientID** | **Gene** | **cDNAVariant** | **CNV** | **ProteinVariant** | **Zygosity** | **ACMG** | **Mono-allelic variant in recessive genes** |
| --- | --- | --- | --- | --- | --- | --- | --- |
| GR0001 | PKD1 | c.1548G>A | No | p.Trp516* | Het | C5 |  |
| GR0002 | PKD1 | c.6871C>T | No | p.Gln2291* | Het | C5 |  |
| GR0004 | PKD2 | c.973C>T | No | p.Arg325* | Het | C5 |  |
| GR0007 | PKD2 | c.2517delT | No | p.Gln840Lysfs*4 | Het | C5 |  |
| GR0011 | PKD2 | c.295G>T | No | p.Glu99* | Het | C5 |  |
| GR0016 | PKD1 | c.4419delC | No | p.Asn1474Met | Het | C5 |  |
| GR0017 | PKD1 | c.3202C>T | No | p.Gln1068* | Het | C5 |  |
| GR0019 | PKD1 | c.10527_10528delGA | No | p.Glu3509Aspfs*117 | Het | C5 |  |
| GR0020 | PKD1 | c.1831C>T | No | p.Arg611Trp | Het | C5 |  |
| GR0022 | PKD1 | c.11294_11313del20 | No | p.Pro3765Argfs*44 | Het | C5 |  |
| GR0023 | PKD1 | c.12061C>T | No | p.Arg4021* | Het | C5 |  |
| GR0024 | PKD1 | c.2702G>A | No | p.Trp901* | Het | C4 |  |
| GR0025 | PKD2 | g.(?_88928831)_(88996847_?)del | Yes |  | Het | C5 |  |
| GR0027 | PKD1 | c.1548G>A | No | p.Trp516* | Het | C5 |  |
| GR0028 | PKD2 | c.878G>A | No | p.Trp293* | Het | C5 |  |
| GR0030 | PKD1 | c.10441delG | No | p.Val3481Serfs*46 | Het | C5 |  |
| GR0031 | PKD1 | c.10725_10726dup | No | p.Val3576Glyfs*10 | Het | C5 |  |
| GR0035 | PKD1 | c.-209-?_2853+?del | Yes |  | Het | C5 |  |
| GR0040 | PKD1 | c.4214G>A | No | p.Trp1405* | Het | C5 |  |
| GR0043 | PKD1 | c.11294_11313del20 | No | p.Pro3765Argfs*44 | Het | C4 |  |
| GR0047 | PKD2 | c.411delG | No | p.Ser138Alafs*95 | Het | C4 |  |
| GR0049 | PKD2 | c.1445delT | No | p.Phe482Serfs*32 | Het | C5 |  |
| GR0050 | PKD2 | c.411delG | No | p.Ser138Alafs*95 | Het | C4 |  |
| GR0052 | PKD1 | c.8284_8295delATCCTCATGCGC | No | p.Ile2762_Arg2765del | Het | C4 |  |
| GR0054 | PKD1 | c.11112_11119dupCATCAAGC | No | p.Gln3707Profs*122 | Het | C5 |  |
| GR0061 | PKD1 | c.10441delG | No | p.Val3481Serfs*46 | Het | C4 |  |
| GR0062 | PKD1 | c.10822-2A>G | No |  | Het | C4 |  |
| GR0063 | PKD1 | c.5014_5015delAG | No | p.Arg1672Gly | Het | C5 |  |
| GR0066 | PKD1 | c.1831C>T | No | p.Arg611Trp | Het | C4 |  |
| GR0067 | PKD2 | c.753delG | No | p.Met252* | Het | C5 |  |
| GR0069 | PKD1 | c.3346C>T | No | p.Gln1116* | Het | C5 |  |
| GR0070 | PKD1 | c.4995delC | No | p.Trp1666Gly | Het | C5 |  |
| GR0071 | PKD2 | c.1807dupA | No | p.Met603Asn | Het | C5 |  |
| GR0072 | PKD1 | c.6643C>T | No | p.Arg2215Trp | Het | C4 |  |
| GR0074 | PKD1 | c.12178C>T | No | p.Gln4060* | Het | C5 |  |
| GR0075 | PKD1 | c.12167G>A | No | p.Trp4056* | Het | C5 |  |
| GR0079 | PKD1 | c.12589_12590delins19 | No | p.Leu4197Met | Het | C4 |  |
| GR0081 | PKD1 | c.7222C>T | No | p.Arg2408Cys | Het | C4 |  |
| GR0085 | COL4A3 | c.2330G>A | No | p.Gly777Asp | Het | C4 |  |
| GR0089 | PKD1 | c.8477_8481dup | No | p.Leu2828Serfs*49 | Het | C5 |  |
| GR0090 | PKHD1 | c.2414C>T | No | p.Pro805Leu | Het | C5 | * |
| GR0091 | PKD2 | c.1094+1del | No |  | Het | C4 |  |
| GR0092 | PKD2 | c.878G>A | No | p.Trp293* | Het | C5 |  |
| GR0103 | PKD1 | c.7292T>A | No | p.Leu2431Gln | Het | C4 |  |
| GR0109 | COL4A3 | c.4981C>T | No | p.Arg1661Cys | Het | C4 |  |
| GR0110 | PKD1 | c.4988_4990del | No | p.Ser1663del | Het | C4 |  |
| GR0112 | PKHD1 | c.9689del | No | p.Asp3230Val | Het | C4 | * |
| GR0119 | PKD1 | c.12673C>T | No | p.Gln4225* | Het | C5 |  |
| GR0128 | PKD2 | c.1807dupA | No | p.Met603Asnfs*23 | Het | C4 |  |
| GR0142 | PKD1 | c.4828_4830delATC | No | p.Ile1610del | Het | C4 |  |
| GR0145 | PKD1 | c.1831C>T | No | p.Arg611Trp | Het | C4 |  |
| GR0159 | PKD1 | c.6611_6612delC | No | p.Val2204Glyfs*57 | Het | C5 |  |
| GR0175 | PKD2 | c.2052C>A | No | p.Tyr684* | Het | C4 |  |
| GR0177 | PKD1 | c.7957_7958del | No | p.Leu2653Glu | Het | C4 |  |
| GR0178 | PKD1 | c.856_862dup | No | p.Gln288Leu | Het | C5 |  |
| GR0185 | PKD1 | c.11156G>A | No | p.Arg3719Gln | Het | C5 |  |
| GR0210 | PKD2 | c.295G>T | No | p.Glu99* | Het | C5 |  |
| GR0223 | PKD2 | c.1094+1del | No |  | Het | C4 |  |
| GR0224 | PKD1 | c.165_171del | No | p.Leu56Argfs*15 | Het | C5 |  |
| MA-0001 | PKD1 | c.6397_6399delTTC | No | p.Phe2133del | Het | C4 |  |
| MA-0003 | PKD1 | c.5501_5502del | No | p.Asn1834Serfs*15 | Het | C5 |  |
| MA-0005 | PKD1 | c.2494dup | No | p.Arg832Profs*40 | Het | C5 |  |
| MA-0006 | PKD2 | c.230dup | No | p.Ala69Glyfs*23 | Het | C5 |  |
| MA-0010 | PKHD1 | c.6170C>A | No | p.Ala2057Asp | Het | C4 | * |
| MA-0012 | PKD1 | c.12010C>T | No | p.Gln4004* | Het | C5 |  |
| MA-0013 | PKHD1 | c.5380+1G>A | No |  | Comp Het | C4 |  |
|  | PKHD1 | c.8388C>G | No | p.Ser2796Arg | Comp Het | C3 |  |
| MA-0019 | PKD1 | c.8578C>T | No | p.Gln2860* | Het | C4 |  |
| MA-0021 | PKD1 | c.74_75delinsT | No | p.Gly25Valfs*48 | Het | C4 |  |
| MA-0023 | PKD2 | c.1681del | No | p.Ile561* | Het | C4 |  |
| MA-0027 | PKD1 | c.5861dupA | No | p.Asn1954Lysfs*36 | Het | C5 |  |
| MA-0028 | PKD1 | c.6827T>C | No | p.Leu2276Pro | Het | C4 |  |
| MA-0029 | PKD2 | c.592C>T | No | p.Arg198* | Het | C4 |  |
| MA-0030 | PKD1 | c.6574_6580del | No | p.Thr2192Alafs*18 | Het | C5 |  |
| MA-0031 | PKD1 | c.10026del | No | p.Leu3343Serfs*54 | Het | C5 |  |
| MA-0032 | PKD2 | c.973C>T | No | p.Arg325* | Het | C5 |  |
| MA-0033 | PKD1 | c.5976_5978del | No | p.Phe1992_Thr1993delinsLeu | Het | C5 |  |
| MA-0034 | PKHD1 | c.737T>C | No | p.Ile246Thr | Comp Het | C4 |  |
|  | PKHD1 | c.5896dup | No | p.Leu1966Thrfs*4 | Comp Het | C4 |  |
| MA-0035 | PKD1 | c.8017-?_8161+? | No |  | Het | C4 |  |
| MA-0036 | PKD2 | c.1774C>T | No | p.Arg592* | Het | C5 |  |
| MA-0039 | PKD1 | c.1261C>T | No | p.Arg421Cys | Het | C4 |  |
| MA-0041 | PKD1 | c.10038del | No | p.Met3346Ilefs*51 | Het | C4 |  |
| MA-0043 | PKD2 | c.1445delT | No | p.Phe482Serfs*32 | Het | C5 |  |
| MA-0044 | PKD2 | c.1445delT | No | p.Phe482Serfs*32 | Het | C5 |  |
| MA-0045 | PKD1 | c.7984C>T | No | p.Gln2662* | Het | C5 |  |
| MA-0047 | PKD1 | c.8973C>G | No | p.Tyr2991* | Het | C5 |  |
| MA-0052 | PKD2 | c.2518C>T | No | p.Gln840* | Het | C4 |  |
| MA-0054 | PKD1 | c.12683G>C | No | p.Arg4228Pro | Het | C4 |  |
| MA-0055 | PKD1 | c.4957C>T | No | p.Gln1653* | Het | C5 |  |
| MA-0056 | PKD2 | c.916C>T | No | p.Arg306* | Het | C5 |  |
| MA-0058 | PKD1 | c.9559_9561del | No | p.Asp3187del | Het | C4 |  |
| MA-0059 | PKD2 | c.2301_2302insA | No | p.Gln768Thrfs*7 | Het | C4 |  |
| MA-0060 | PKD1 | c.10686C>A | No | p.Ser3562Arg | Het | C4 |  |
| MA-0061 | PKD2 | c.7540del | No | p.Leu2514Cysfs*106 | Het | C4 |  |
| MA-0066 | PKD2 | c.916C>T | No | p.Arg306* | Het | C5 |  |
| MA-0067 | PKD1 | c.10952G>C | No | p.Gly3651Ala | Het | C4 |  |
| MA-0068 | PKD1 | c.11871dupT | No | p.Ala3958Cysfs*3 | Het | C4 |  |
| MA-0071 | PKD1 | c.6593del | No | p.Pro2198Argfs*14 | Het | C4 |  |
| MA-0072 | PKD1 | c.9683dupG | No | p.Leu3229Profs*24 | Het | C5 |  |
| MA-0081 | PKD2 | c.2358+1G>T | No |  | Het | C5 |  |
| MA-0087 | PKD2 | c.1094+3_1094+6del | No |  | Het | C5 |  |
| MA-0088 | PKD1 | c.1144G>C | No | p.Gly382Arg | Het | C4 |  |
| MA-0090 | PKD2 | c.1445delT | No | p.Phe482Serfs*32 | Het | C5 |  |
| MA-0091 | PKD1 | c.2152C>T | No | p.Gln718* | Het | C5 |  |
| MA-0092 | PKD1 | c.4349_4351del | No | p.Asn1450del | Het | C4 |  |
| MA-0093 | PKD2 | c.648C>A | No | p.Tyr216* | Het | C4 |  |
| MA-0094 | PKD2 | c.2191C>T | No | p.Gln731* | Het | C4 |  |
| MA-0095 | PKD2 | c.1848C>G | No | p.Tyr616* | Het | C5 |  |
| MA-0098 | PKD2 | c.965G>A | No | p.Arg322Gln | Mosaicism | C5 |  |
| MA-0105 | PKD1 | c.2985+1G>A | No |  | Het | C5 |  |
| MA-0106 | PKD2 | c.991T>C | No | p.Cys331Arg | Het | C4 |  |
| MO-0002 | PKD1 | c.11215C>T | No | p.Gln3739* | Het | C5 |  |
| MO-0006 | PKD1 | c.8860G>T | No | p.Glu2954* | Het | C5 |  |
| MO-0008 | PKD1 | c.4092dupC | No | p.Val1365Argfs*66 | Het | C5 |  |
| MO-0012 | PKD2 | c.2614C>T | No | p.Arg872* | Het | C5 |  |
| MO-0021 | PKD1 | c.4306C>T | No | p.Arg1436* | Het | C5 |  |
| MO-0044 | PKD1 | c.5014_5015delAG | No | p.Arg1672Glyfs*98 | Het | C5 |  |
| MO-0062 | PKD2 | c.1960C>T | No | p.Arg654* | Het | C5 |  |
| MO-0063 | PKD1 | c.9202-10_9218del | No |  | Het | C5 |  |
| MO-0066 | PKD1 | c.9397+1G>A | No |  | Het | C5 |  |
| MO-0069 | PKHD1 | c.9524A>G | No | p.Asn3175Ser | Comp Het | C4 |  |
|  | PKHD1 | c.10484T>C | No | p.Leu3495Pro | Comp Het | C4 |  |
| MO-0113 | PKD2 | c.2117del | No | p.Lys706Argfs*10 | Het | C5 |  |
| MO-0125 | PKD1 | c.1577A>C | No | p.His526Pro | Het | C4 |  |
| MO-0129 | PKD1 | c.12445-34_12445-10del | No |  | Het | C4 |  |
| MO-0143 | PKD1 | c.10035del | No | p.Met3346Cysfs*51 | Het | C5 |  |
| MO-0157 | PKD1 | c.7546C>T | No | p.Arg2516Cys | Het | C4 |  |
| MO-0161 | PKD1 | c.211G>C | No | p.Ala71Pro | Het | C4 |  |
| MO-0162 | PKD1 | c.6730_6731del | No | p.Ser2244Hisfs*17 | Het | C5 |  |
| MO-0164 | PKD1 | c.8251C>T | No | p.Gln2751* | Het | C5 |  |
| MO-0165 | PKD1 | c.7300C>T | No | p.Arg2434Trp | Het | C4 |  |
| MO-0179 | PKD1 | c.6184C>T | No | p.Gln2062* | Het | C5 |  |
| MO-0181 | PKD1 | c.8704C>T | No | p.Gln2902* | Het | C5 |  |
| MO-0182 | PKD1 | c.8704C>T | No | p.Gln2902* | Het | C5 |  |
| MO-0184 | COL4A5 | c.878_879del | No | p.Glu293Alafs*6 | Het | C4 |  |
| MO-0187 | PKD1 | c.(8016+1_8017-1)_(11016+1_11017-1)del | Yes |  | Het | C5 |  |
| MO-0188 | PKD1 | c.8311G>A | No | p.Glu2771Lys | Het | C5 |  |
| MO-0208 | PKD1 | c.5875del | No | p.Ala1959Profs*13 | Het | C5 |  |
| MO-0209 | PKD1 | c.8311G>A | No | p.Glu2771Lys | Het | C5 |  |
| MO-0211 | PKHD1 | c.7328T>G | No | p.Leu2443* | Comp Het | C5 |  |
|  | PKHD1 | c.7264T>G | No | p.Cys2422Gly | Comp Het | C4 |  |
| MO-0244 | PKD2 | c.1319+1G>A | No |  | Het | C5 |  |
| MO-0245 | PKD2 | c.1319+1G>A | No |  | Het | C5 |  |
| MO-0307 | PKHD1 | c.(880+1_881-1)_(976+1_977-1)del | No |  | Het | C4 | * |
| MO-0315 | PKD1 | c.11340_11346dup | No | p.Val3783Leufs*35 | Het | C5 |  |
| MO-0332 | PKD1 | c.6084C>G | No | p.Tyr2028* | Het | C4 |  |
| MO-0348 | PKHD1 | c.6731T>A | No | p.Leu2244His | Homo | C4 |  |
| MO-0361 | PKD1 | c.12691C>T | No | p.Gln4231* | Het | C5 |  |
| MO-0362 | PKD1 | c.12453_12454del | No | p.His4151Glnfs*5 | Het | C4 |  |
| MO-0386 | PKD2 | c.2296G>T | No | p.Gly766* | Het | C5 |  |
| MO-0389 | PKD1 | c.6760G>T | No | p.Glu2254* | Het | C5 |  |
| MO-0427 | PKD1 | c.9894G>A | No | p.Trp3298* | Het | C5 |  |
| MO-0437 | PKD1 | c.12061C>T | No | p.Arg4021* | Het | C5 |  |
| PR-0001 | PKD1 | c.11172G>A | No | p.Trp3724* | Het | C4 |  |
| PR-0010 | PKD1 | c.12112del | No | p.Val4038* | Het | C4 |  |
| PR-0011 | PKD1 | c.11249G>A | No | p.Arg3750Gln | Het | C5 |  |
| PR-0012 | PKD1 | c.7292T>C | No | Leu2431Pro | Het | C4 |  |
| PR-0013 | PKD2 | c.1158T>A | No | p.Tyr386* | Het | C4 |  |
| PR-0014 | PKD2 | c.1249C>T | No | p.Arg417* | Het | C5 |  |
| PR-0015 | PKD1 | c.11156G>A | No | p.Arg3719Gln | Het | C4 |  |
| PR-0016 | PKD1 | c.12445-1G>T | No |  | Het | C4 |  |
| PR-0017 | PKD1 | c.12184_12185del | No | p.Leu4062Valfs*94 | Het | C4 |  |
| PR-0018 | PKD1 | c.6609_6627del | No | p.Val2204Thrfs*2 | Het | C4 |  |
| PR-0019 | PKD2 | c.637C>T | No | p.Arg213* | Het | C5 |  |
| PR-0002 | PKD1 | c.6487C>T | No | p.Arg2163* | Het | C5 |  |
| PR-0020 | PKD2 | c.1960C>T | No | p.Arg654* | Het | C5 |  |
| PR-0022 | PKD1 | c.2396_2421del | No | p.Arg799Glnfs*9 | Het | C5 |  |
| PR-0024 | PKD1 | c.1295C>T | No | p.Ala432Val | Het | C4 |  |
| PR-0027 | PKD1 | c.7415_7416insA | No | p.Ser2475Leufs*26 | Het | C4 |  |
| PR-0028 | PKD2 | c.2208_2213delAAACTT | No | p.Leu736_Asn737del | Het | C4 |  |
|  | PKHD1 | c.8824C>T | No | p.Arg2942* | Het | C5 |  |
| PR-0029 | PKD1 | c.6142del | No | p.Ser2048Valfs*68 | Het | C4 |  |
| PR-0003 | PKD1 | c.24_27dup | No | p.Ala10Profs*105 | Het | C4 |  |
| PR-0031 | PKD2 | c.1774C>T | No | p.Arg592* | Het | C5 |  |
| PR-0033 | PKD2 | c.637C>T | No | p.Arg213* | Het | C5 |  |
| PR-0034 | PKD1 | c.4888C>T | No | p.Gln1630* | Het | C5 |  |
| PR-0035 | PKD1 | c.6184C>T | No | p.Gln2062* | Het | C5 |  |
| PR-0036 | PKD1 | c.7546C>T | No | p.Arg2516Cys | Het | C5 |  |
| PR-0037 | PKD1 | c.5003_5022delinsGCC | No | p.Ala1668Glyfs*97 | Het | C4 |  |
|  | PKHD1 | c.8870T>C | No | p.Ile2957Thr | Het | C5 |  |
| PR-0038 | PKD2 | Gene deletion | Yes |  | Het | C5 |  |
| PR-0004 | PKD1 | c.9157G>A | No | p.Ala3053Thr | Het | C4 |  |
| PR-0005 | PKD1 | c.11441_11442del | No | p.Tyr3814* | Het | C4 |  |
| PR-0006 | PKD2 | c.1717-1G>T | No |  | Het | C4 |  |
| PR-0007 | PKD2 | c.2614C>T | No | p.Arg872* | Het | C5 |  |
| PR-0009 | PKD1 | c.3597_3598del | No | p.Ala1200Glyfs*10 | Het | C4 |  |

*CNV*, copy number variation; *ACMG*, American College of Medical Genetics and Genomics; *Het,* Heterozygous; *Comp Het*, Compound Heterozygous; *Homo*, Homozygous, *?,* variant annotated from the readings that cover the exon region and the closest flanking nucleotides (+/- 25 bp).

* Note: Biallelic pathogenic variants are required for a definitive autosomal recessive diagnosis; monoallelic findings are provisional and may warrant additional CNV or deep-intronic analyses. Further details in the previous section ‘Diagnostic Implications of Single Heterozygous Variants in Autosomal-Recessive Kidney Disorders’ of this supplementary material.

**Table S5:** Diagnostic variants in patients with Glomerulopathy.

| **Patient ID** | **Gene** | **cDNAVariant** | **CNV** | **ProteinVariant** | **Zygosity** | **ACMG** | **Monoallelic variant in recessive genes** |
| --- | --- | --- | --- | --- | --- | --- | --- |
| GR0076 | COL4A5 | c.3605G>A | No | p.Gly1202Asp | Het | C4 |  |
| GR0080 | COL4A5 | c.2605G>A | No | p.Gly869Arg | Hemi | C5 |  |
| GR0086 | COL4A5 | c.1843G>A | No | p.Gly615Arg | Het | C5 |  |
| GR0087 | COL4A5 | c.440dupG | No | p.Pro148Thr | Hemi | C5 |  |
| GR0094 | COL4A4 | c.941G>A | No | p.Gly314Asp | Het | C4 |  |
| GR0096 | COL4A3 | c.343G>A | No | p.Gly115Arg | Het | C4 |  |
| GR0115 | COL4A3 | c.40_63del | No | p.Leu14_Leu21del | Het | C4 |  |
| GR0121 | COL4A4 | c.941G>A | No | p.Gly314Asp | Het | C4 |  |
| GR0127 | CYP24A1 | c.1226T>C | No | p.Leu409Ser | Het | C5 |  |
| GR0131 | COL4A4 | c.482G>A | No | p.Gly161Asp | Het | C4 |  |
| GR0134 | COL4A5 | c.3605G>A | No | p.Gly1202Asp | Het | C4 |  |
| GR0155 | COL4A5 | c.3605G>A | No | p.Gly1202Asp | Het | C4 |  |
| GR0163 | COL4A3 | c.4421T>C | No | p.Leu1474Pro | Het | C4 |  |
| GR0169 | COL4A3 | Exon 12 del | Yes |  | Het | C4 |  |
| GR0170 | COL4A3 | c.4421T>C | No | p.Leu1474Pro | Het | C4 |  |
| GR0184 | COL4A4 | c.1108G>A | No | p.Gly370Arg | Het | C4 |  |
| GR0196 | COL4A3 | c.4421T>C | No | p.Leu1474Pro | Het | C4 |  |
| GR0205 | COL4A5 | c.3508G>A | No | p.Gly1170Ser | Het | C5 |  |
| MA-0014 | COL4A5 | c.395G>A | No | p.Gly132Glu | Het | C5 |  |
| MA-0084 | COL4A4 | c.4932delCinsTT | No | p.Ala1645Cysfs*3 | Het | C5 |  |
| MO-0001 | COL4A4 | c.1889delC | No | p.Pro630Glnfs*23 | Het | C5 |  |
| MO-0003 | COL4A5 | c.1424-4C>G | No |  | Het | C5 |  |
| MO-0026 | COL4A5 | c.3088G>A | No | p.Gly1030Ser | Het | C4 |  |
| MO-0033 | COL4A3 | c.1363G>T | No | p.Gly455Cys | Het | C4 |  |
| MO-0034 | COL4A3 | c.1363G>T | No | p.Gly455Cys | Het | C4 |  |
| MO-0043 | COL4A3 | c.1363G>T | No | p.Gly455Cys | Het | C4 |  |
| MO-0049 | COL4A5 | c.1424-4C>G | No |  | Het | C5 |  |
| MO-0064 | COL4A5 | c.973G>A | No | p.Gly325Arg | Het | C5 |  |
| MO-0065 | COL4A3 | c.2083G>A | No | p.Gly695Arg | Het | C4 |  |
| MO-0083 | COL4A5 | c.448G>C | No | p.Gly150Arg | Het | C4 |  |
| MO-0087 | PKD1 | c.9499A>T | No | p.Ile3167Phe | Het | C4 |  |
| MO-0099 | COL4A5 | c.5020C>T | No | p.Arg1674* | Het | C5 |  |
|  | CYP24A1 | c.1421del | No | p.Leu474Trpfs*102 | Het | C5 |  |
| MO-0101 | COL4A3 | c.4928+1G>A | No |  | Comp Het | C5 |  |
|  | COL4A3 | c.4421T>C | No | P.Leu1474Pro | Comp Het | C4 |  |
| MO-0114 | COL4A4 | c.2951del | No | p.Gly972Glufs*66 | Het | C5 |  |
|  | CUBN | c.6928_6934del | No |  | Het | C5 |  |
| MO-0126 | COL4A3 | c.4235G>T | No | p.Gly1412Val | Het | C4 |  |
| MO-0130 | COL4A5 | c.1075G>A | No | p.Gly359Arg | Het | C4 |  |
| MO-0139 | COL4A5 | c.3688G>A | No | p.Gly1223Asp | Het | C4 |  |
| MO-0144 | COL4A4 | c.2320G>C | No | p.Gly774Arg | Comp Het | C4 |  |
|  | COL4A4 | c.4394G>A | No | p.Gly1465Asp | Comp Het | C4 |  |
| MO-0168 | TTC21B | c.626C>T | No | p.Pro209Leu | Homo | C5 |  |
| MO-0198 | COL4A3 | c.4421T>C | No | p.Leu1474pro | Het | C4 |  |
| MO-0199 | COL4A5 | c.2282del | No | Pro761Hisfs*31 | Het | C5 |  |
| MO-0210 | COL4A5 | c.2282del | No | p.Pro761Hisfs*31 | Het | C5 |  |
| MO-0215 | COL4A4 | c.189_192+256del | No |  | Het | C4 |  |
| MO-0241 | COL4A4 | c.1423G>C | No | p.Gly475Arg | Het | C4 |  |
| MO-0265 | FN1 | c.1155G>A | No | p.Trp385* | Het | C5 |  |
|  | COL4A3 | c.765G>A | No |  | Het | C5 |  |
| MO-0292 | CUBN | c.(4168+1_4169-1)_(4350+1_4351-1)del | Yes |  | Het | C4 |  |
| MO-0329 | COL4A5 | c.3731G>A | No | p.Gly1244Asp | Het | C5 |  |
| MO-0344 | COL4A5 | c.448G>C | No | p.Gly150Arg | Het | C4 |  |
| MO-0390 | COL4A5 | c.2282del | No | p.Pro761Hisfs*31 | Het | C5 |  |
| MO-0395 | COL4A3 | c.4421T>C | No | p.Leu1474Pro | Het | C4 |  |
| MO-0398 | COL4A4 | c.104A>G | No | p.Tyr35Cys | Comp Het | C4 |  |
|  | COL4A4 | c.5048G>A | No | p.Cys1683Tyr | Comp Het | C4 |  |
| MO-0441 | COL4A3 | c.2083G>A | No | p.Gly695Arg | Het | C4 |  |

*CNV*, copy number variation; *ACMG*, American College of Medical Genetics and Genomics; *Het*, Heterozygous; *Comp Het*, Compound Heterozygous; *Homo*, Homozygous; *Hemi*, Hemizygous.

* Note: Biallelic pathogenic variants are required for a definitive autosomal recessive diagnosis; monoallelic findings are provisional and may warrant additional CNV or deep-intronic analyses. Further details in the section ‘Diagnostic Implications of Single Heterozygous Variants in Autosomal-Recessive Kidney Disorders’ of this supplementary material.

**Table S6:** Diagnostic variants in patients with CAKUT

| **PatientID** | **Gene** | **cDNAVariant** | **CNV** | **ProteinVariant** | **Zygosity** | **ACMG** | **Monoallelic variant in recessive genes** |
| --- | --- | --- | --- | --- | --- | --- | --- |
| MO-0041 | PAX2 | c.410+5G>A | No |  | Het | C4 |  |
| MO-0045 | HNF1B | c.908G>A | No | p.Arg303His | Het | C4 |  |
| MO-0298 | HNF1B | c.162del | No | p.Glu55Serfs*70 | Het | C4 |  |
| MO-0375 | HNF1B | g.36047280_36104883del | Yes |  | Het | C5 |  |
| MO-0397 | SIX1 | c.243del | No | p.Lys82Asnfs*7 | Het | C5 |  |
| MO-0429 | PAX2 | c.76dup | No | p.Val26Glyfs*28 | Het | C5 |  |

*CAKUT*, congenital abnormalities of kidney and urinary tract; *CNV*, copy number variation; *ACMG*, American College of Medical Genetics and Genomics; *Het*, Heterozygous.

* Note: Biallelic pathogenic variants are required for a definitive autosomal recessive diagnosis; monoallelic findings are provisional and may warrant additional CNV or deep-intronic analyses. Further details in the section ‘Diagnostic Implications of Single Heterozygous Variants in Autosomal-Recessive Kidney Disorders’ of this supplementary material.

**Table S7:** Diagnostic variants in patients with Nephrolithiasis.

| **PatientID** | **Gene** | **cDNAVariant** | **CNV** | **ProteinVariant** | **Zygosity** | **ACMG** | **Monoallelic variant in recessive genes** |
| --- | --- | --- | --- | --- | --- | --- | --- |
| MO-0256 | CYP24A1 | c.1186C>T | No | p.Arg396Trp | Het | C4 | * |
| MO-0297 | CYP24A1 | c.428_430del | No | p.Glu143del | Het | C4 | * |
| MO-0371 | CYP24A1 | c.428_430del | No | p.Glu143del | Het | C4 | * |
| MO-0421 | CYP24A1 | c.1226T>C | No | p.Leu409Ser | Het | C5 | * |

*CNV*, copy number variation; *ACMG*, American College of Medical Genetics and Genomics; *Het*, Heterozygous.

* Note: Biallelic pathogenic variants are required for a definitive autosomal recessive diagnosis; monoallelic findings are provisional and may warrant additional CNV or deep-intronic analyses. Further details in the section ‘Diagnostic Implications of Single Heterozygous Variants in Autosomal-Recessive Kidney Disorders’ of this supplementary material.

**Table S8:** Diagnostic variants in patients with Tubulopathy.

| **PatientID** | **Gene** | **cDNAVariant** | **CNV** | **ProteinVariant** | **Zygosity** | **ACMG** | **Monoallelic variant in recessive genes** |
| --- | --- | --- | --- | --- | --- | --- | --- |
| GR0095 | UMOD | c.947A>C | No | p.Gln316Pro | Het | C4 |  |
| GR0214 | SLC12A3 | c.2089_2095del | No | p.Thr697Glyfs*2 | Het | C5 | * |
| MA-0002 | SLC12A3 | c.852+1G>A | No |  | Het | C5 | * |
| MO-0115 | SLC12A3 | c.644T>C | No | p.Leu215Pro | Comp Het | C4 |  |
|  | SLC12A3 | c.1390G>A | No | p.Ala464Thr | Comp Het | C4 |  |
| MO-0116 | SLC4A4 | c.654del | No | p.Lys218Asnfs*31 | Het | C5 | * |
| MO-0266 | ATP6V0A4 | c.1185del | No | p.Tyr396Thrfs*12 | Het | C5 | * |
| MO-0315 | GRHPR | c.905G>A | No | p.Arg302His | Het | C4 | * |
| MO-0360 | SLC12A3 | c.3052C>T | No | p.Arg1018* | Comp Het | C5 |  |
|  | SLC12A3 | c.812T>C | No | p.Leu271Pro | Comp Het | C3 |  |

*CNV*, copy number variation; *ACMG*, American College of Medical Genetics and Genomics; *Het*, Heterozygous; *Comp Het*, Compound Heterozygous.

* Note: Biallelic pathogenic variants are required for a definitive autosomal recessive diagnosis; monoallelic findings are provisional and may warrant additional CNV or deep-intronic analyses that are not currently available in this case. See the “Diagnostic Implications of Monoallelic Variants in Autosomal-Recessive Kidney Disorders” section of this Supplementary Material for full details.

**Table S9:** Predictive model evaluation score based on the test set (accuracy, area under the receiver operating characteristic curve (AUC), F1 score, precision, recall, and specificity).

|  | **Value** |
| --- | --- |
| Predictions: | |
| Correct - diagnostic variant (True Positive) | 54/208 |
| Incorrect - diagnostic variant (False Negative) | 44/208 |
| Correct - not a diagnostic variant (True Negative) | 88/208 |
| Incorrect - not a diagnostic variant (False Positive) | 22/208 |
| Metrics: | |
| Accuracy (%) | 68 |
| TPR (%) - sensitivity | 55 |
| TNR (%) - specificity | 80 |
| AUC | 0.78 |
| Precision | 0.71 |
| F1 score | 0.62 |

*TPR*, true positive rate; *TNR*, true negative rate; *AUC*, area under the curve.

**Table S10:** Reclassification of the initial clinical suspicion, after genetic testing.

|  | **Clinical diagnosis** | **Genetic diagnosis** | **Zygosity** | **Definitive diagnosis** |
| --- | --- | --- | --- | --- |
| 1 | IgAN | COL4A3 | Het | Alport syndrome |
| 2 | Interstitial nephritis | COL4A3 | Het | Alport syndrome |
| 3 | Glomerulopathy of unknown origin, KF | TTC21B | Homo | Nephronophtisis |
| 4 | Glomerulosclerosis | PAX2 | Het | Renal Coloboma Syndrome |
| 5 | Tubulopathy not defined | HNF1b | Het | HNF1b-CAKUT |
| 6 | Glomerulopathy of unknown origin | PKD1 | Het | ADPKD |

*KF*, Kidney failure; *Het*, Heterozygous; *CAKUT*, congenital abnormalities of kidney and urinary tract; ADPKD, Autosomal Dominant Polycystic Kidney Disease.
